# A Case-Control Study on the Changes in High-Sensitivity C-Reactive Protein and Tumor Necrosis Factor-Alpha Levels with Surgical Treatment of OSAS

**DOI:** 10.3390/ijms232214116

**Published:** 2022-11-15

**Authors:** Ewa Olszewska, Tymoteusz Marek Pietrewicz, Magdalena Świderska, Jacek Jamiołkowski, Adrian Chabowski

**Affiliations:** 1 Department of Otolaryngology, Medical University of Bialystok, 15-089 Bialystok, Poland; 2 Department of Physiology, Medical University of Bialystok, 15-089 Bialystok, Poland; 3 Department of Population Medicine and Lifestyle Diseases Prevention, Medical University of Bialystok, 15-089 Bialystok, Poland

**Keywords:** sleep apnea, inflammation, cytokines, sleep surgery

## Abstract

Obstructive sleep apnea syndrome (OSAS) is a common but underdiagnosed condition with significant health and economic implications for society. Inflammatory mediators are proposed to be associated with the presence and severity of OSAS and contribute to morbidity and mortality. This paper details a prospective non-randomized case control study of a cohort of subjects, who underwent surgical treatment of OSAS and were enrolled to assess the sleep parameters and blood levels of selected inflammatory markers at pre-operative and post-operative time points, also comparing them to the levels in a control group. A total of 25 study subjects and 18 control subjects were enrolled. Median values and interquartile range (IQR) of the apnea–hypopnea index (AHI) in the study group pre-operatively and post-operatively were 34 (18.5–45.5) and 13.3 (7.5–27.3), while in the control group 1.4 (1.0–2.1) per hour. The mean (IQR) hs-CRP levels (mg/L) were 1.782 (0.941–5.594) and 1.980 (0.990–5.445) in the study group, pre-operatively and post-operatively, respectively, while 0.891 (0.767–1.436) in the control group. The mean (IQR) TNF-α levels (pg/mL) were 7.999 (6.137–9.216) and 6.614 (5.534–7.460) pre-and post-operatively, respectively, and were 6.000 (5.026–6.823) in the control group. Results demonstrated that both inflammatory markers, hs-CRP and TNF-α, are higher in subjects with OSAS compared to the controls, and their levels decrease, but are still higher than the controls, after successful surgical treatment. Further analysis including the body mass index and age demonstrated that these changes were significant for TNF-α, but not hs-CRP.

## 1. Introduction

Obstructive sleep apnea syndrome (OSAS) is a common ailment with an increasing frequency of occurrence. Due to health, society, and economic effects, it is crucial to find factors that could improve diagnostic procedures for this disease. OSAS is a chronic inflammatory disease with cyclic episodes of partial or total obstruction of upper respiratory airways during sleep with preserved respiratory muscle effort [1]. Decreased upper respiratory muscle tone during sleep leads to episodes of reduced airflow. A fluctuation of blood oxygen saturation level occurs leading to hypoxic–hypercapnic periods during sleep [2]. The pathomechanism of vascular endothelial dysfunction in patients with OSAS has not been well established yet. The endothelium is an active tissue that lines vascular walls, functions as a barrier, and regulates arterial blood pressure by secreting biologically active substances. Studies have shown that repeated hypoxia episodes result in oxidative stress in the tissues [3], leading to the release of free oxygen radicals and an increase in the secretion of pro-inflammatory factors, including tumor necrosis factor-alpha (TNF-α), high sensitivity C-reactive protein (hs-CRP), and interleukin 6 (IL-6) [4].

hs-CRP actively participates in the formation of atherosclerotic plaques through the induction and expression of adhesive particles. The influence of IL-6 and TNF-α elevates the blood hs-CRP concentration, whose main function is to maintain homeostasis. IL-6 is a cytokine secreted by activated macrophages and lymphocytes. IL-6 production is stimulated by inflammatory conditions such as smoking and excess fatty tissues. Factors affecting hs-CRP concentration are age, gender, and smoking [5]. Adipose tissue, particularly visceral fat, is the site where IL-6 synthesis occurs, leading to increased hs-CRP levels, particularly in obese people. Due to both the systemic and local inflammatory characteristics of OSAS, many scientists showed a correlation between the severity of OSAS, expressed as obstructive apnea–hypopnea index (AHI), and the hs-CRP levels in the blood sera of these patients [6]. Other studies also revealed an association between the blood hs-CRP concentration and the presence of OSAS as well as a decrease in the blood hs-CRP levels after the treatment of OSAS [7]. Because of inflammatory factors within the atherosclerotic plaques, hs-CRP is considered a marker of future potential cardiovascular adverse outcomes and dysfunction in endothelial integrity [8]. Studies demonstrated hs-CRP as a significant risk factor for atherosclerosis, myocardial infarction, and stroke [9].

An increase in the blood serum TNF-α levels was observed in the OSAS patients [10]. Studies on rats demonstrated that the administration of exogenous TNF-α results in a prolonged mainly non-rapid eye movement (NREM)-sleep phase and increased free wave amplitudes in the electroencephalographic (EEG) recordings [11]. TNF-α leads to NF-κB pathway activation, which activates the receptors of nitrogen oxide synthase, cyclo-oxygenase 2, and adenosine A1, which are all involved in sleep regulation [12]. Elevated TNF-α concentrations in OSAS and obesity may be playing a role in both the pathogenesis and consequences of both conditions [13]. Etanercept therapy, a preparation inhibiting TNF-α secretion, has been shown to decrease the AHI and daytime somnolence [14].

The goal of the study was to assess the impact of OSAS on the level of selected inflammatory markers, such as hs-CRP and TNF-α. The specific aim was to evaluate the effect of surgical treatment of OSAS patients on hs-CRP and TNF-α concentrations in blood. We aimed to assess whether the concentration of hs-CRP and TNF-α in the blood is higher in OSAS patients compared to the controls, which would indicate that OSAS promotes inflammation. We also assessed whether blood TNF- α and hs-CRP levels in OSAS patients are higher compared to the control group, and whether their levels decrease after the surgical treatment of OSAS.

## 2. Results

### 2.1. Characteristics of Groups Based on Age, Gender, BMI, and Laryngological Examination

The median age in the group at enrollment was 48 years, with a mean of 50.2 ± 11.3 years in the control group, and the median age was 33 years. There was a significant difference between age in the pre-treatment group and the control group (*p* < 0.0001). Four females (16%) and 21 males (84%) were included in the study group before the surgery while there were 10 females (55.6%) and 8 males (44.4%) in the control group. 

Based on the National Institute of Health guidelines [15], the mean BMI in the study group before surgery indicated class 1 obesity (34.1 ± 3.7). BMI at the time of post-operative assessments had reduced to 31.7 ± 3.7. In the control group, the mean BMI was categorized as overweight (25 ± 5.4). There was a significant difference between BMI in the pre-treatment group and the post-treatment group and BMI in the control group (*p* < 0.005). On the other hand, there was no significant difference between BMI in the group before the treatment and BMI in the post-treatment group (*p* = 0.062). In the pre-treatment group, 4% of patients had normal BMI, 24% were overweight, 56% were class 1 obese and 16% were class 2 obese. In the control group, 50% of patients had normal BMI, 44.4% were overweight and 5.6% were class 3 obese.

In the evaluation of the size of the palatal tonsils according to Friedman, class 3 obesity was found in 16 patients, class 2 was found in 9 patients.

Based on Friedman’s staging system [16], in the evaluation of the size of the base of the tongue, Friedman grade IIa was found in two patients (8%), grade IIb in eight patients (32%), grade III in twelve patients (48%) and grade IV in three patients (12%).

### 2.2. Type of Surgery

In the study group, the following was performed:

- 10 modified uvulopalatopharyngoplasty (modified U3P) treatments with tongue base tissue reduction;
- 10 modified U3P procedures with radiofrequency induced thermotherapy of the tongue base;
- 5 expansion sphincter pharyngoplasty (ESP) treatments with tongue base tissue reduction.

### 2.3. OSAS Severity, ODI, MOS, LOS

Comparing the results in the pre-operative group and the post-operative group, there was an improvement in the polygraph test for the apnea/hypopnea index (AHI) within the study group. A 60.9% reduction in median AHI was observed after the surgical procedure. There was a significant difference between the median of the pre-operative group and the median of the control group (*p* < 0.005), as well as between the median of the pre-operative group and the median of the post-operative group (*p* < 0.005). There was a significant difference between the median of the post-operative group and the median of the control group (*p* < 0.005). The AHI value significantly decreased after surgery; however, it did not improve to the level in the control group. There was an improvement in the oxygen saturation index (ODI) with the surgery, while ODI in both the pre-operative and post-operative groups was different from the controls. A 48.3% reduction in the median after the surgical procedure was obtained. There was a significant difference between the median of the pre-operative group and the median of the control group (*p* < 0.005), as well as between the median of the pre-operative group and the median of the post-operative group (*p* < 0.005). There was a significant difference between the median of the post-operative group and the median of the control group (*p* < 0.005). Comparing the results in the pre-operative group and the post-operative group, there was an improvement in the polygraph test for mean oxygen saturation (MOS). A 1.4% increase in the median was obtained after the surgical procedure. There was a significant difference between the median of the pre-operative group and the median of the control group (*p* < 0.005), as well as between the median of the pre-operative group and the median of the post-operative group (*p* = 0.007). There was a significant difference between the median of the post-operative group and the median of the control group (*p* < 0.005). Comparing the results in the pre-operative group and the post-operative group, there was an improvement in the polygraph test for lowest oxygen saturation (LOS). A median increase of 9.3% was obtained after the surgical procedure. There was a significant difference between the median of the pre-operative group and the median of the control group (*p* < 0.005), as well as between the median of the pre-operative group and the median of the post-operative group (*p* = 0.005). There was a significant difference between the median of the post-operative group and the median of the control group (*p* < 0.005). The summary of AHI, ODI, MOS, and LOS results is presented in Table 1.

### 2.4. ESS, VAS, SF-36

We also observed an improvement in the score on Epworth Sleepiness Scale (ESS). Comparing the results in the pre-operative group and the post-operative group, an improvement in the results on the ESS was obtained. In the pre-surgery group, the median ESS questionnaire score was 13 points and the inter-quartile range (IQR) was 8.5–19; while in the post-surgery group, the median score was 5 points (IQR: 2–7.5). In the control group, the median score was 3 points (IQR: 1–5.3). A 61.5% reduction in median score after the surgical procedure was achieved. There was a significant difference between the median in the pre-operative group and the median in the control group (*p* < 0.005), as well as between the median in the pre-operative group and the median in the control group (*p* < 0.005). There was no significant difference between the median of the post-operative group and the median of the control group (*p* = 0.116). A 61.5% reduction in the median was achieved. The level of daytime sleepiness in the post-surgery group was not significantly different from the control group.

There was an improvement in visual scale of snoring (VAS) scores regarding the severity of snoring annoyance among patients who underwent surgery. In the pre-surgery group, the median score was 10 points (IQR: 9–10); and in the post-surgery group, the median score was 4 points (IQR: 2.5–7.0). In the control group, the median result was 2 points (IQR: 1–3.3). A 60% reduction in median score was achieved after the surgical procedure. There was a significant difference between the median of the pre-operative group and the median of the control group (*p* < 0.005), as well as between the median of the pre-operative group and the median of the post-operative group (*p* < 0.005). There was a significant difference between the median of the post-operative group and the median of the control group (*p* = 0.007).

Improvements were obtained in the scores of a questionnaire assessing the quality of life, utilizing the 36-item Short Form Health Survey (SF-36) instrument. In the pre-operative group, the median result was 72 points (IQR: 52–81.5); and in the post-operative group, the median score was 36 points (IQR: 23.5–45). In the control group, the median score was 43.5 points (IQR: 29–59). A 50% reduction in the median score was achieved after the surgical procedure. There was a significant difference between the median of the pre-operative group and the median of the control group (*p* < 0.005), as well as between the median of the pre-operative group and the median of the post-operative group (*p* < 0.005). There was no significant difference between the median of the post-operative group and the median of the control group (*p* = 0.099).

### 2.5. hs-CRP

**Figure 1:**
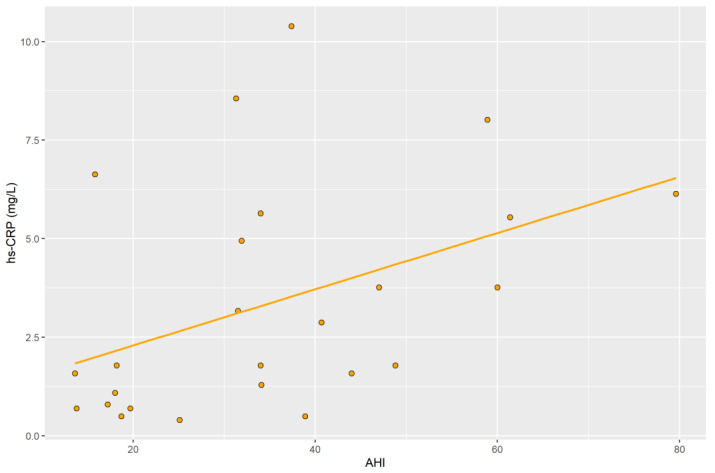
Correlation of AHI with hs-CRP levels in the pre-operative group.

The median serum hs-CRP level obtained from the blood of patients in the study group before surgery was 1.782 mg/L (IQR: 0.941–5.594); whereas, in the post-operative group, the median was 1.980 mg/L (IQR: 0.990–5.445). In the control group, the median was 0.891 mg/L (IQR: 0.767–1.436) (Table 2). A significant difference was obtained between the median concentration in the pre-operative group and the control group (*p* = 0.033), and a significant difference was obtained in the median concentration between the post-operative group and the control group (*p* = 0.043). No significant difference was obtained in the median concentration between the pre-surgery group and the post-surgery group (*p* = 0.855). In the pre-operative group, there was a positive correlation between AHI and serum hs-CRP levels obtained from the blood of patients in this group (r = 0.42; *p* = 0.035) (Figure 1). Analyzing the parameters after the surgery, there were no significant correlations between hs-CRP and BMI (*p* = 0.75, r = 0.07), and between hs-CRP and AHI (*p* = 0.24, r = 0.27).

The median in the pre-operative group and the post-operative group was significantly higher as compared to the median in the control group.

### 2.6. TNF-α

The median serum TNF-α level obtained from the blood of patients in the pre-operative group was 7.999 pg/mL (IQR: 6.137–9.216); whereas, in the post-operative group, the median was 6.614 pg/mL (IQR: 5.534–7.460). In the control group, the median concentration was 6.000 pg/mL (IQR: 5.026–6.823) (Table 2). A significant difference was obtained between the median in the pre-operative group and the median in the control group (*p* < 0.005). A significant difference was obtained between the median in the pre-operative group and the median in the post-operative group (*p* = 0.002). However, no statistically significant difference was obtained between the median of the group after surgery and the median in the control group (*p* = 0.218).

A 17.3% reduction in median TNF-α concentration was obtained after the surgical procedure. The difference in median serum TNF-α levels obtained from the patient’s blood before and after surgery was not significant.

### 2.7. Generalized Estimating Equations

Generalized estimating equations (GEE) models were used to analyze additional effects of body weight and age changes on TNF-α and hs-CRP concentrations (Table 3). This analysis did not demonstrate a significant effect of change in the BMI and age on the hs-CRP levels. On the other hand, this model demonstrated a significant change for the TNF-α. For TNF-α, we have a significant effect of the surgery: a significant value of factor B = −1.175 means that after the surgery the value of TNF is mean lower than 1.175 u. We also have a significant effect of BMI; B = 0.143 means that a participant with higher BMI by 1 unit has an expected value of TNF higher by 0.143 units.

## 3. Discussion

Evaluation of inflammatory marker concentrations in blood is considered for assessment of the degree of a generalized inflammatory condition [17]. In a recent study, patients with OSAS revealed elevated levels and activation of pro-inflammatory factors such as TNF-α [18]. The authors also indicated that elevated levels of proteins mentioned above are associated with increased cardiovascular morbidity. Coronary heart disease, hypertension, arrhythmia, and congestive heart failure are among the cardiovascular diseases in which OSAS has been identified as an independent risk factor [19].

In our results, in the group of patients with OSAS before surgery, TNF-α levels were significantly higher compared to those in the control group. Similar results were obtained by Ming et al. who found higher levels of TNF-α in the blood of patients with obstructive sleep apnea syndrome compared to the control group [20]. This observation is also consistent with the results of several other studies [21,22,23]. Moreover, TNF-α levels were shown to be reduced after the PAP treatment [24]. In a systematic review including 19 studies, Nadeem et al. presented significantly higher levels of TNF-α in serum obtained from the blood of OSAS patients compared to controls [17]. Similarly, in a Wang et al. publication, which analyzed seven studies in 2015, serum TNF-α levels were also significantly higher in patients with OSAS [25]. Cao et al., based on a meta-analysis published in 2020, showed that TNF-α levels in the blood of patients with OSAS are 1.77 times higher compared to TNF-α levels in patients without this disease [10]. Results in the above-mentioned publications are consistent with the results of our research. However, Fornadi et al., in a study on a group of 100 patients with OSAS after kidney transplantation, did not confirm higher levels of TNF-α in the blood of these patients [26]. According to the authors of this study, the use of immunosuppressive drugs in the study group may have influenced the lack of correlation of inflammatory marker levels with sleep disorders. The influence of diurnal rhythm on the secretion of cytokines and some hormones were the foci of several publications. Entzian et al., in 1996, showed that TNF-α significantly affects fatigue and sleepiness in patients with OSAS [27]. Similarly, Vgontzas et al. presented an association between elevated TNF-α blood levels and excessive daytime sleepiness. They hypothesized that this cytokine is involved in the regulation of physiological sleep and that higher levels of TNF-α are related to sleepiness and fatigue [13]. Therefore, it is reasonable to monitor TNF-α blood levels in patients with OSAS. The correlation between ESS and elevated TNF-α blood levels in OSAS patients is considered an important parameter in the assessment of these patients. However, in the current study, we did not demonstrate a positive correlation between TNF-α and daytime sleepiness expressed on the ESS scale. This may be due to the smaller sample size in our study. It has been shown that continuous PAP therapy can inhibit TNF-α secretion. In addition, OSAS patients who received anti-TNF-α therapy had a significant reduction in daytime sleepiness and AHI scores [14]. Comparing the results in pre-and post-surgery in the study group, we showed a significant decrease in the TNF-α levels. Our results were consistent with the findings of Kataoka et al. [28].

In our study, there was no positive correlation between the degree of disease progression, expressed in the AHI and serum TNF-α levels. The opposite conclusion was reached by Li et al. who showed a positive correlation of TNF-α concentration with the degree of disease progression [29]. We also did not find a positive correlation between TNF-α levels and BMI. Similarly, in a study on a group of 65 patients with OSAS, Ciftci et al. did not show a correlation between the BMI value and TNF-α concentrations in the blood of patients with OSAS [21]. Furthermore, we did not observe positive correlations between TNF-α levels and hs-CRP levels in the blood of patients with OSAS. Zumbach et al. demonstrated that TNF-α stimulates leptin secretion, whose effects were associated with sympathetic nervous system activation and increased blood pressure [30]. Blake et al. found in their study that TNF-α is an important factor in the development of atherosclerotic lesions within arteries, leading to leukocyte adhesion to the vascular endothelium [31]. Skoog et al. also observed a positive correlation between levels of this cytokine and signs of early atherosclerosis in a group of 96 young men [32]. Ridker et al. in two publications confirmed that elevated TNF-α levels in patients after myocardial infarction increase the risk of subsequent coronary events [18,33]. Increased concentration of TNF-α in the blood is associated with an increased risk of metabolic and cardiovascular diseases. The concentration of pro-inflammatory cytokines, TNF-α, in the blood is elevated in OSAS and obesity. It may play a role in the pathogenesis and consequences of both conditions [13]. Ryan et al. in 2006 found that TNF-α is also a marker of cardiovascular diseases in OSAS [34]. It seems that evaluation of cardiovascular disease risk in OSAS patients based on TNF-α blood levels in OSAS patients would be a valuable complement to the previous studies.

Human and animal studies suggest the role of TNF-α in regulating respiration. Reid et al. in 2002 published a study demonstrating the effects of TNF-α on pharyngeal muscle dysfunction [35]. Li et al. demonstrated, in their study on mice, that eight-week exposure to circulating TNF-α at clinically relevant concentrations (250–350 pg/mL) caused oxidative stress and contractile dysfunction in the mouse diaphragm [36]. Elevated TNF-α levels are associated with a higher risk of metabolic and cardiovascular disease.

Analysis of our study indicated significantly higher serum hs-CRP levels in patients with OSAS as compared to patients in the control group. Similar findings were also made by Sharma et al. in a study of a group of 52 patients [37] published in 2008. Moreover, other studies also obtained higher serum hs-CRP levels in OSAS patients [38,39].

In our study, significantly higher levels of hs-CRP in blood sera obtained from OSAS patients were found in both the pre-operative group and the post-operative group, as compared with the levels of this protein in the blood of patients in the control group. This may be due to the fact that there was no complete recovery (as AHI < 5) in the post-operative sleep study. Higher hs-CRP concentrations in OSAS patients, compared to controls, have been shown in two others meta-analyses, which is reflected in the results of the present study [17,40].

In our study, we obtained positive correlations between hs-CRP and disease severity, expressed by AHI, and quality of life score, expressed by the SF-36 scale, in a group of patients before surgery, independent of obesity. The correlations between hs-CRP with BMI and the AHI were not significant both before and after the surgical treatment. The correlations between TNF-α with BMI and the AHI were not significant both before and after the surgical treatment. In the available literature, we did not find a clear answer to the correlations of blood hs-CRP levels with OSAS severity. In 2020, Kang et al. published a meta-analysis based on nine papers involving 277 patients. The authors found significant reductions in AHI and serum hs-CRP levels in patients undergoing surgical treatment for OSAS. The authors did not obtain a positive correlation between serum hs-CRP concentration and OSAS severity, expressed in AHI [41]. Shamsuzzaman et al., in a 2002 study, described a positive correlation between hs-CRP concentration and AHI indicator. The authors found that patients with prolonged sleep fragmentation have higher serum hs-CRP concentrations than those in the controls [42]. However, Ryan et al. obtained different results; they showed that blood hs-CRP concentrations in male patients without diagnosed OSAS are similar to the concentrations of this protein in the blood of patients with mild, moderate, and severe OSAS [34]. A significant increase in blood hs-CRP levels was observed in obese patients with severe OSAS. It was found that hs-CRP levels did not correlate with the severity of OSAS while a positive correlation with obesity was demonstrated, which is not consistent with the results of our study [34].

The results of the research paper by Testelmans et al. are consistent with the results of our study, as the authors demonstrated a positive correlation between OSAS severity and hs-CRP levels [43]. Numerous studies indicate an important role played by hs-CRP in the pathogenesis of atherosclerotic changes and cardiovascular diseases [42,44]. Researchers suggest that hs-CRP activates inflammation at the vascular level and may play a role in disease pathogenesis. It is a factor of the inflammatory condition that occurs in atherosclerosis [45].

A study by Lui et al. has shown a positive correlation of hs-CRP levels with disease stage/severity expressed in AHI [46]. However, they are not in agreement with the studies of Sharma et al. and Akashiba et al., which did not show evidence of a correlation of hs-CRP with OSAS [37,47]. The Wisconsin Sleep Cohort Study project involving 907 patients also found no independent association between hs-CRP levels and OSAS severity as expressed in AHI. In a 2021 meta-analysis, based on a review of 44 studies, Imani et al. found significantly higher serum hs-CRP levels in patients with OSAS as compared with controls [48]. Lee et al. evaluated 65 patients newly diagnosed with different severity of OSAS. They measured a serum level of hs-CRP in this group of study participants and concluded that the AHI was an independent predictor of increasing serum level of this protein. The authors stated that the hs-CRP concentration level of ≥3 mg/L is prevalent in the severe OSAS group, therefore it is recommended to measure the serum levels of this protein in this group and prioritize them for treatment to limit cardiovascular complications [49]. Further study by the same author and the team measured serum levels of this protein in a group of 30 patients with OSA before and 6 months after relocation pharyngoplasty. They observed a significant decrease in hs-CRP concentration in serum after the surgery; however, it was related to very severe OSAS patients with AHI ≥ 60. Overall, in 40% of the whole OSA group, a noticeable decrease in the serum level was observed [50].

Factors influencing hs-CRP levels may include age and gender [5]. Garcia et al. in a 2016 study on a group of patients with risk factors for metabolic syndrome, published results showing significantly higher serum hs-CRP levels obtained from the blood of females as compared to serum levels in a group of males [51]. BMI is also important in OSAS patients who underwent surgery because it may affect hs-CRP levels. Our study did not show a positive correlation between hs-CRP and BMI in these patients. This is consistent with the results obtained by Jung et al., who showed no positive correlation between blood hs-CRP levels and BMI in patients with OSAS [52]. However, Guilleminault et al. believe that hs-CRP levels in OSAS patients positively correlated with body mass index and this was the only parameter that independently correlated with elevated blood hs-CRP levels in OSAS patients included in this study [53].

It is important to emphasize the fact that hs-CRP is a crucial inflammatory marker in serum. This protein is regulated by IL-6 expression which, according to Imani et al. studies, is increased in OSAS patients as compared to the controls [48]. Bouloukaki et al. found that elevated levels of hs-CRP are observed in individuals with cardiovascular disease risk factors, such as atherosclerosis, stroke, and myocardial infarction [9,54]. The connections between the thickness of carotid intima-media complex and hs-CRP concentrations were analyzed by Minoguchi et al., who showed that the studied parameters were significantly higher in the group of OSAS compared to healthy controls [55]. Studies indicate that increased serum hs-CRP levels may predict cardiovascular complications such as atherosclerosis [54]. Taking that into account, prolonged sustained hypoxia may cause activation of the inflammatory response with elevated levels of proinflammatory cytokines [56]. Patients with increased visceral fat have increased inflammation within the adipose tissue. Few studies have addressed the impact of OSAS on inflammation and how much it is potentiated by the effects of proinflammatory cytokines secreted within visceral fat in obese patients.

In the control group, we obtained lower hs-CRP levels, which may indicate a lower risk of cardiovascular events. However, in the group of patients diagnosed with OSAS, hs-CRP levels were significantly higher. The control determination of this inflammatory marker did not show that the surgery performed significantly influenced the change in serum hs-CRP levels. Kheirandish-Gozal et al. found that elevated hs-CRP levels predispose to a higher risk of cardiovascular diseases, diabetes, and cognitive decline [57]. Research directions aim to determine the feasibility of preventing cardiovascular complications in patients with OSAS based on the measurement of blood hs-CRP levels. In conclusion, elevated serum hs-CRP levels are associated with OSAS, and may reflect an increased risk of cardiovascular morbidity. Based on our results, we found that the concentration of hs-CRP in serum, which is an independent risk factor for cardiovascular diseases, is significantly higher in the group of patients with OSAS. The elevated blood levels of hs-CRP may be one of the mechanisms responsible for the cardiovascular complications associated with OSAS.

It is conceivable that weight could be considered an independent variable with respect to the inflammatory markers measured in the study. The changes in the BMI and age during the interval between the two study measurements, at the pre-operative and post-operative assessments, could potentially have been responsible for the changes in the inflammatory marker levels. Even though the GEE analysis did not demonstrate such an effect for the hs-CRP, this could be due to the small sample size. On the other hand, this more in-depth analysis, taking into account the influence of BMI and age, demonstrated a significant change for TNF-α.

While our study results discussed above indicated major differences in the inflammatory marker levels between the study group (pre-and post-operative) and the control group, such a comparison should have controlled for the variables that may have independently affected the results. As explained in the methods and study limitations sections, the control group is different from the study group for the two major risk factors for OSAS, old age, and high BMI. A control group with age and BMI similar to the study group but without OSAS would have been preferred, if possible, for a more accurate comparison between the groups. This would have demonstrated better the impact of age and BMI, independent of the presence of OSAS, and the potential value of these markers. Future studies with larger sample sizes and/or with a balanced distribution of such variables may be necessary to derive more definitive conclusions.

## 4. Materials and Methods

### 4.1. Study Protocol

Twenty-five adult patients were prospectively included in the study group. All patients underwent planned surgical treatment for OSAS. All the surgical procedures were performed by the manuscript’s first author (EO). The local Bioethical Committee’s approval No. 61/2016 was obtained for the study. Patients gave written informed consent for a study protocol including the assessments, tests, and surgical treatment.

Inclusion criteria:

- adult patients, between the ages of 18–70 years
- OSAS diagnosed based on polygraphic examination (sleep study type III), following the guidelines of the American Academy of Sleep Medicine, medical history, and physical examination;
- lack of tolerance of PAP therapy
- no surgical treatment for snoring or OSAS minimum 6 months prior to the enrollment;
- written informed consent to participate in the study

Exclusion criteria were: facial skeleton abnormalities, neoplastic diseases/malignancy, acute infection, chronic inflammatory diseases, chronic renal disorders, enteritis, chronic liver diseases, uncontrolled diabetes, respiratory and circulatory failure, pregnancy, use of anti-inflammatory medications, and lack of written consent for the participation in the study.

The prospectively enrolled control group consisted of patients scheduled for stapedotomy, without evidence of sleep-disordered breathing (SDB), such as snoring, apnea, or hypopnea, and did not suffer from any chronic, immunological, or neoplastic diseases. Control patients also gave written informed consent for the study tests and procedures. The control group met the same inclusion and exclusion criteria, except for the qualifying characteristic of the presence of OSAS in the study group.

Before the surgery, each subject underwent a trial of PAP treatment (positive airway pressure) as per the eligibility for enrollment. On admission, subjects in both the study and control groups were informed of the study protocols and methods. Medical history was obtained. Body weight and height measurements were taken and body mass index (BMI) was calculated. Under the diagnostic protocol in OSAS, a complete laryngological examination including an evaluation of upper airways was conducted with the use of a nasofiberoscope. The morphology of upper airways was assessed, including the size of the tongue, palatal tonsils, soft palate and uvula, posterior pharyngeal wall, laryngopharynx, and the structures of the larynx. The evaluation of nasal mucous membranes, nasal septum alignment, and the size of nasal turbinates was conducted. Co-existing diseases and previous treatments were evaluated. The patients completed specific sleep questionnaires including the assessment of the quality of life using a 36-item Short Form Health Survey (SF-36), and the evaluation of excessive daytime somnolence using the Epworth Sleepiness Scale (ESS). Each patient rated the degree of their annoyance with snoring with the visual scale of snoring (VAS) instrument, with 0 being the least annoyance and 10 being the maximum annoyance. The polygraphic examination was performed with Embletta MPR (Embletta MPR, Middleton, USA), to obtain sleep parameters and for the diagnosis of OSAS. Following the AASM definition, the severity of the disease was determined based on the AHI index (apnea/hypopnea index) [58]. In addition to AHI, the following parameters were evaluated: AHI, ODI (oxygen desaturation index), MOS (mean oxygen saturation), and LOS (lowest oxygen saturation).

All the study assessments (except the pre-operative sleep study) were performed within 2 weeks before and 9 ± 2 months after the surgical treatment in the study group. The pre-operative sleep study was performed within 4 months before the surgery.

Before the surgery anteroposterior (A/P) chest X-ray, and electrocardiography were obtained. In addition, to evaluate as per routine pre-operative workup for general anesthesia and to rule out chronic and inflammatory disease, the following biochemical blood tests were analyzed before and after the surgical treatment: peripheral blood morphology, sodium, potassium, aspartate aminotransferase (AST), alanine transaminase (ALT), creatinine, urea, glucose, activated partial thromboplastin (APTT), prothrombin time, and calcium clotting time.

In the control group, all pre-operative assessments were performed identically to the study group in the surgery for otosclerosis.

### 4.2. Determination of hs-CRP and TNF-α in Serum

Blood was collected from the subjects also, to obtain serum for the evaluation of selected protein concentrations. A fasting blood sample was collected on the morning of the procedure. A total of 15 mL of venous blood was centrifuged at 2000 rpm for 10 min at +4 °C, and obtained serum was stored at −80 °C until the assays were performed. Measurement of the selected protein concentrations was carried out in the same laboratory, by the same person, using the ELISA method. Two protein levels were measured hs-CRP and TNF-α, by the manufacturer’s assay protocol.

hs-CRP concentration was measured using the hs-CRP, HS (C-Reactive Protein) (EIA-3954) ELISA kit (DRG, Marburg, Germany). The assay was performed using a BioTek reader and Gen5 software using a standard 96-well plate coated with a monoclonal mouse antibody (anti-hs-CRP) directed against hs-CRP antigen and horseradish peroxidase with dissolved goat anti-hs-CRP antibody. By using the two antibodies, the detected hs-CRP antigen molecule was placed between the solid phase in the well and the enzyme-bound tetramethylbenzidine (TMB) reagent. The blue color of the reagents was obtained with the incubation and the color was changed into yellow by adding 1N HCl. The concentration of hs-CRP was directly proportional to the color intensity of the test sample. Absorbance was measured spectrophotometrically at 450 nm. The lowest detection threshold was 0.1 mg/L hs-CRP and the highest detection threshold was 10 mg/L hs-CRP.

TNF-α concentration was obtained with the use of the ELISA TNF-α test, EIA-4641 (DRG, Marburg, Germany). A monoclonal antibody (Mab) on a microplate was used in the assay, directed against different TNF-α epitopes, monoclonal antibody 2 (Mab 2), and horseradish peroxidase (HRP). After incubation, a so-called sandwich was formed (MAb1-TNF-α-MAb 2-HRP detected).

An automatic washer was used for rinsing between reactions for best results. The process was finished with 1.8N H2SO4. Afterward, colorimetric reading was conducted by the measurement of the absorbance, proportional to TNF-α concentration. A BioTek reader with a wavelength of 450 nm and 490 nm was used.

### 4.3. Surgical Procedures

All the subjects in the study group underwent the surgical procedure within the oropharynx under general anesthesia. The surgical treatment consisted of single-stage surgery at two levels of the upper airways, expansion sphincterpharyngoplasty or modified uvulopalatopharyngoplasty (modified U3P) at the soft palate level and the coblation of the tongue base.

Repeat blood draws and assays on the serum after the surgery in the study group were performed concurrent with the pre-operative samples, as outlined in the study protocol.

### 4.4. Statistical Analysis

In statistical analysis, due to a limited number of samples, non-parametric methods were used. Mann–Whitney U tests were used to compare the study and the control groups for quantitative analysis. Wilcoxon pairwise rank order tests were used to assess differences between dependent samples (assessing differences before and after treatment). Correlation relationships between pairs of quantitative variables were described using Spearman’s rank correlation coefficients. All statistical hypotheses were verified at a significance level of α = 0.05. Statistical calculations were performed using IBM SPSS Statistics software (SPSS Inc., Chicago, IL, USA) version 26.0.

A total of 43 patients were analyzed and divided into three groups:

Study groups:

Group 1—before surgery: 25 patients (4 females, 21 males) met the inclusion criteria and did not meet the exclusion criteria of the study.

Group 2—after surgery: same 25 patients from group 1 after surgery.

Control group:

Group 3: 18 patients qualified for stapedotomy (10 females and 8 males).

## 5. Conclusions

1. OSAS promotes an inflammatory state.
2. Surgical treatment reduces inflammation expressed by TNF-α levels in OSAS patients.

The TNF-α and hs-CRP concentrations in the blood of patients with OSAS are higher compared to the control group.

Further studies may contribute to the diagnostic and therapeutic algorithm based on the determination of hs-CRP and TNF-α concentrations.

## 6. Study Limitations

1. The main limitation of our study was the size of the study group (*n* = 25 patients). Despite our best efforts, of the 35 patients included, only 25 of them showed up for follow-up. The limited sample was also due to the need to eliminate factors that could have influenced the results (including unregulated diabetes, enteritis, thyroid diseases, and chronic liver diseases). Due to time limitations, resulting from the period of reagents used for the determinations of concentration of particular compounds, it was difficult to obtain a fully homogeneous control group, that met the inclusion, and did not meet the exclusion criteria of the study. Moreover, not all patients included in the study group consented to repeat blood sampling after surgery.
2. The control group, who were selected from patients undergoing surgery for otosclerosis, was quite different with respect to age and BMI than the study group, who were selected from those that needed sleep surgery due to the presence of OSAS. Although minimally invasive, the burden due to the tests and samples required for the study enrollment as a control subject limited the potentially eligible population. Due to relative convenience, and to reduce the burden, control subjects were selected among those that needed surgery, but also did not have OSAS. This limited further the ability to find control group candidates with age and BMI similar to the study subjects with older age and BMI who needed sleep surgery associated with such factors. Lower age range and BMI in the control group limited their true role as control when such major variables were not comparable to the study group. Those factors may independently impact the levels of inflammatory markers. In addition, the condition of the control group that qualified the enrollment was otosclerosis disease, which is also considered an inflammatory condition. Even though there are reports on the presence of increased expression of TNF-α in the histological samples of the stapes footplate, we did not find literature indicating elevated levels of these markers in the serum. However, we cannot completely rule out the potential effect of otosclerosis or any other inflammatory conditions in the subjects in the control group on the levels of TNF-α and hs-CRP.
3. Based on the literature, the difference in the magnitude of BMI in the study group and the control group may affect the results of the study. Nevertheless, analysis of the blood concentrations of selected compounds in patients before and after surgery indicates a lack of significant change in BMI values in these groups of patients. Thus, we suppose that the influence of the factor related to the change in body weight on the results of our study was small.
4. The levels of the inflammatory markers in the blood of patients after surgery may have been influenced by lifestyle, diet, and physical activity in the period between determinations.
5. The results of our study were not compared to the results of the study carried out on a group of patients undergoing non-surgical treatment.
6. In our experiment we used type III sleep study (polygraphy) in the diagnostic procedure. During polygraphy, sleep structure and arousal identification are not analyzed. However, according to the American Academy of Sleep Medicine, the results of this examination allow qualifying patients for surgery, especially when the study group does not include patients with coexisting diseases [58].
7. The polygraph examination also has advantages, which include, among others, that it was conducted in a home setting, which increased the chance of sleep undisturbed by hospital conditions. The report was not automatically generated. In each case, a manual reading of the sleep record by the first and second authors was used to obtain this report.
8. Due to a number of factors, although all done within 4 months before the surgery, there was variability in the interval between the sleep study and the surgical treatment, therefore authors cannot rule out a possible change in the severity of the sleep study results during that interval.

## Figures and Tables

**Table 1:** Results as mean values and standard deviation (SD) and median values with interquartile range (IQR) of the apnea–hypopnea index (AHI), oxygen saturation index (ODI), mean oxygen saturation (MOS), and lowest oxygen saturation (LOS) in the pre-operative group, post-operative group, and control group.

Sleep Parameters	Before Surgery	After Surgery	Control
	Mean ± SD [Median (IQR)]	Mean ± SD [Median (IQR)]	Mean ± SD [Median (IQR)]
Number of Subjects (*n*)	25	25	18
AHI	34.9 ± 17.3 [34 (18.5–45.5)]	18.7 ± 15.3 [13.3 (7.5–27.3)]	1.5 ± 0.8 [1.4 (1.0–2.1)]
ODI	37.2 ± 18.2 [35 (22.3–47.6)]	22.6 ± 17.6 [18.1 (9.8–29.7)]	1.9 ± 0.8 [1.9 (1.2–2.5)]
MOS (%)	91.3 ± 2.9 [91.6 (91.0–92.8)]	92.6 ± 2.2 [92.9(91.7–93.7)]	95.6 ± 1.0 [95.9 (95.0–96.1)]
LOS (%)	74.2 ± 11.8 [75.0 (68.5–82.0)]	80.1 ± 8.3 [82.0 (75.5–86.0)]	88.4 ± −5.4 [89.5 (87.0–92.3)]

**Table 2:** Results as mean values and standard deviation (SD) and median values with interquartile range (IQR) of high sensitivity C-reactive protein (hs-CRP) and tumor necrosis factor-alpha (TNF- α) in the pre-operative group, post-operative group, and control group.

Inflammatory Markers	Before Surgery	After Surgery	Control
	Mean ± SD [Median (IQR)]	Mean ± SD [Median (IQR)]	Mean ± SD [Median (IQR)]
Number of Subjects (*n*)	25	25	18
hs-CRP (mg/L)	3.356 ± 2.879 [1.782 (0.941–5.594)]	3.334 ± 3.160 [1.980 (0.990–5.445)]	1.775 ± 2.299 [0.891 (0.767–1.436)]
TNF-α (pg/mL)	8.378 ± 3.267 [7.999 (6.137–9.216)]	6.635 ± 1.390 [6.614 (5.534–7.460)]	6.119 ± 1.779 [6.000 (5.026–6.823)]

**Table 3:** Results of the generalized estimating equations (GEE) models to assess the effect of change in the BMI and age on the marker levels after the surgery is presented as estimates, confidence interval (C.I.) and the level of significance (*p*).

Dependent Variable	Independent Variable	B (95% C.I.)	*p*
hs-CRP (mg/L)	After vs. before surgery	0.008 (−0.899–−0.916)	0.985
	Patients vs. controls	2.347 (−1.288–−5.981)	0.206
	BMI	0.306 (−0.090–−0.703)	0.130
	Age	0.137 (−0.051–−0.324)	0.153
TNF-α (pg/mL)	After vs. before surgery	−1.175 (−2.344–−0.005)	0.049 *
	Patients vs. controls	−1.081 (−3.352–−1.190)	0.351
	BMI	0.143 (0.007–−0.279)	0.039 *
	Age	0.006 (−0.063–−0.074)	0.868

*: Statistically significant.

## Data Availability

The data presented in this study are available on request from the corresponding authors. The data are not publicly available.

## Abbreviations

AASM	American Academy of Sleep Medicine
AHI	apnea/hypopnea index
BMI	body mass index
C.I.	confidence interval
ELISA	enzyme-linked immunosorbent assay
ESP	expansion sphincter pharyngoplasty
ESS	Epworth Sleep Scale
hs-CRP	high-sensitivity C-Reactive Protein
IL-6	interleukin 6
IQR	inter-quartile range
LOS	lowest oxygen saturation
modified U3P	modified uvulopalatopharyngoplasty
MOS	mean oxygen saturation
ODI	oxygen saturation index
OSAS	obstructive sleep apnea syndrome
*p*	level of statistical significance
PAP	positive airway pressure
r	correlation coefficient
SD	standard deviation
SDB	sleep-disordered breathing
SF-36	36-item Short Form Health Survey
TNF-α	tumor necrosis factor α
VAS	visual scale of snoring
vs.	versus

## References

[1] Heatley E.M., Harris M., Battersby M., McEvoy R.D., Chai-Coetzer C.L., Antic N.A. (2013). Obstructive sleep apnoea in adults: A common chronic condition in need of a comprehensive chronic condition management approach. Sleep Med. Rev..

[2] Prabhakar N.R., Peng Y.-J., Nanduri J. (2020). Hypoxia-inducible factors and obstructive sleep apnea. J. Clin. Investig..

[3] Olszewska E., Rogalska J., Brzóska M. (2021). The Association of Oxidative Stress in the Uvular Mucosa with Obstructive Sleep Apnea Syndrome: A Clinical Study. J. Clin. Med..

[4] Lavie L. (2015). Oxidative stress in obstructive sleep apnea and intermittent hypoxia—Revisited—The bad ugly and good: Implica-tions to the heart and brain. Sleep Med Rev..

[5] Navarro S.L., Kantor E.D., Song X., Milne G.L., Lampe J.W., Kratz M., White E. (2016). Factors Associated with Multiple Biomarkers of Systemic Inflammation. Cancer Epidemiol. Biomark. Prev..

[6] Boudjeltia K.Z., van Meerhaeghe A., Doumit S., Guillaume M., Cauchie P., Brohée D., Vanhaeverbeek M., Kerkhofs M. (2006). Sleep Apnoea-Hypopnoea Index Is an Independent Predictor of High-Sensitivity C-Reactive Protein Elevation. Respiration.

[7] Tauman R., O’Brien L.M., Gozal D. (2006). Hypoxemia and obesity modulate plasma C-reactive protein and interleukin-6 levels in sleep-disordered breathing. Sleep Breath..

[8] Nilsson J. (2005). CRP—Marker or maker of cardiovascular disease?. Arterioscler. Thromb. Vasc. Biol..

[9] Bouloukaki I., Mermigkis C., Kallergis E.M., Moniaki V., Mauroudi E., E Schiza S. (2015). Obstructive sleep apnea syndrome and cardiovascular disease: The influence of C-reactive protein. World J. Exp. Med..

[10] Cao Y., Song Y., Ning P., Zhang L., Wu S., Quan J., Li Q. (2020). Association between tumor necrosis factor alpha and obstructive sleep apnea in adults: A meta-analysis update. BMC Pulm. Med..

[11] Kubota T., Li N., Guan Z., Brown R.A., Krueger J.M. (2002). Intrapreoptic microinjection of TNF-α enhances non-REM sleep in rats. Brain Res..

[12] Rockstrom M.D., Chen L., Taishi P., Nguyen J.T., Gibbons C.M., Veasey S.C., Krueger J.M. (2018). Tumor necrosis factor alpha in sleep regulation. Sleep Med. Rev..

[13] Vgontzas A.N., Papanicolaou D.A., Bixler E.O., Kales A., Tyson K., Chrousos G.P. (1997). Elevation of plasma cytokines in disorders of excessive daytime sleepiness: Role of sleep disturbance and obesity. J. Clin. Endocrinol. Metab..

[14] Vgontzas A.N., Zoumakis E., Lin H.-M., Bixler E.O., Trakada G., Chrousos G.P. (2004). Marked Decrease in Sleepiness in Patients with Sleep Apnea by Etanercept, a Tumor Necrosis Factor-? Antagonist. J. Clin. Endocrinol. Metab..

[15] National Institutes of Health, National Heart, Lung, and Blood Institute (1998). Clinical guidelines on the identification, evaluation, and treatment of overweight and obesity in adults–the evidence report. Obes. Res..

[16] Friedman M., Salapatas A.M., Bonzelaar L.B. (2017). Updated Friedman Staging System for Obstructive Sleep Apnea. Sleep Related Breathing Disorders.

[17] Nadeem R., Molnar J., Madbouly E.M., Nida M., Aggarwal S., Sajid H., Naseem J., Loomba R. (2013). Serum Inflammatory Markers in Obstructive Sleep Apnea: A Meta-Analysis. J. Clin. Sleep Med..

[18] Ridker P.M., Rifai N., Pfeffer M., Sacks F., Lepage S., Braunwald E. (2000). Elevation of Tumor Necrosis Factor-α and Increased Risk of Recurrent Coronary Events After Myocardial Infarction. Circulation.

[19] Patel N., Donahue C., Shenoy A., Patel A., El-Sherif N. (2016). Obstructive sleep apnea and arrhythmia: A systemic review. Int. J. Cardiol..

[20] Ming H., Tian A., Liu B., Hu Y., Liu C., Chen R., Cheng L. (2018). Inflammatory cytokines tumor necrosis factor-α, interleukin-8 and sleep monitoring in patients with obstructive sleep apnea syndrome. Exp. Ther. Med..

[21] Ciftci T.U., Kokturk O., Bukan N., Bilgihan A. (2004). The relationship between serum cytokine levels with obesity and obstructive sleep apnea syndrome. Cytokine.

[22] De la Peña Bravo M., Serpero L.D., Barceló A., Barbé F., Agustí A., Gozal D. (2007). Inflammatory proteins in patients with obstructive sleep apnea with and without daytime sleepiness. Sleep Breath..

[23] Sahlman J., Miettinen K., Peuhkurinen K., Seppä J., Peltonen M., Herder C., Punnonen K., Vanninen E., Gylling H., Partinen M. (2010). The activation of the inflammatory cytokines in overweight patients with mild obstructive sleep apnoea: Sleep apnea and inflammation. J. Sleep Res..

[24] Dorkova Z., Petrasova D., Molcanyiova A., Popovnakova M., Tkacova R. (2008). Effects of Continuous Positive Airway Pressure on Cardiovascular Risk Profile in Patients With Severe Obstructive Sleep Apnea and Metabolic Syndrome. Chest.

[25] Wang J., Yu W., Gao M., Zhang F., Gu C., Yu Y., Wei Y. (2015). Impact of obstructive sleep apnea syndrome on endothelial function, arterial stiffening, and serum inflammatory markers: An updated meta-analysis and metaregression of 18 studies. J. Am. Heart Assoc..

[26] Fornadi K., Lindner A., Czira M.E., Szentkiralyi A., Lazar A., Zoller R., Turanyi C.Z., Veber O., Novak M., Mucsi I. (2011). Lack of association between objectively assessed sleep disorders and inflammatory markers among kidney transplant recipients. Int. Urol. Nephrol..

[27] Entzian P., Linnemann K., Schlaak M., Zabel P. (1996). Obstructive sleep apnea syndrome and circadian rhythms of hormones and cytokines. Am. J. Respir. Crit. Care Med..

[28] Kataoka T., Enomoto F., Kim R., Yokoi H., Fujimori M., Sakai Y., Ando I., Ichikawa G.I., Ikeda K. (2004). The effect of surgical treatment of obstructive sleep apnea syndrome on the plasma TNF-α levels. Tohoku J. Exp. Med..

[29] Li Y., Chongsuvivatwong V., Geater A., Liu A. (2009). Exhaled breath condensate cytokine level as a diagnostic tool for obstructive sleep apnea syndrome. Sleep Med..

[30] Zumbach M.S., Boehme J., Wahl P., Stremmel W., Ziegler R., Nawroth P.P. (2015). Tumor necrosis factor increases serum leptin levels in humans. J. Clin. Endocrinol. Metab..

[31] Blake G.J., Ridker P.M. (2002). Inflammatory bio-markers and cardiovascular risk prediction. J. Intern. Med..

[32] Skoog T., Dichtl W., Boquist S., Skoglund-Andersson C., Karpe F., Tang R., Bond M., De Faire U., Nilsson J., Eriksson P. (2002). Plasma tumour necrosis factor-α and early carotid atherosclerosis in healthy middle-aged men. Eur. Heart J..

[33] Ridker P.M., Paynter N.P., Rifai N., Gaziano J.M., Cook N.R. (2008). C-reactive protein and parental history improve global cardiovas-cular risk prediction: The Reynolds risk score for men. Circulation.

[34] Ryan S., Nolan G.M., Hannigan E., Cunningham S., Taylor C., McNicholas W.T. (2007). Cardiovascular risk markers in obstructive sleep apnoea syndrome and correlation with obesity. Thorax.

[35] Reid M.B., Lännergren J., Westerblad H. (2002). Respiratory and limb muscle weakness induced by tumor necrosis factor-α: Involvement of muscle myofilaments. Am. J. Respir Crit. Care Med..

[36] Li X., Moody M.R., Engel D., Walker S., Clubb F.J., Sivasubramanian N., Mann D.L., Reid M.B. (2000). Cardiac-Specific Overexpression of Tumor Necrosis Factor-α Causes Oxidative Stress and Contractile Dysfunction in Mouse Diaphragm. Circulation.

[37] Sharma S.K., Mishra H.K., Sharma H., Goel A., Sreenivas V., Gulati V., Tahir M. (2008). Obesity, and not obstructive sleep apnea, is re-sponsible for increased serum hs-CRP levels in patients with sleep-disordered breathing in Delhi. Sleep Med..

[38] Chung S., Yoon I.-Y., Shin Y.-K., Lee C.H., Kim J.-W., Lee T., Choi D.-J., Ahn H.J. (2007). Endothelial Dysfunction and C-Reactive Protein in Relation with the Severity of Obstructive Sleep Apnea Syndrome. Sleep.

[39] Firat Guven S., Turkkani M.H., Ciftci B., Ulukavak Ciftci T., Erdogan Y. (2012). The relationship between high-sensitivity C-reactive protein levels and the severity of obstructive sleep apnea. Sleep Breath..

[40] Van der Touw T., Andronicos N.M., Smart N. (2019). Is C-reactive protein elevated in obstructive sleep apnea? A systematic review and meta-analysis. Biomarkers.

[41] Kang K., Yeh T., Hsu Y., Ko J., Lee C., Lin M., Hsu W. (2020). Effect of Sleep Surgery on C-Reactive Protein Levels in Adults With Obstructive Sleep Apnea: A Meta-Analysis. Laryngoscope.

[42] Shamsuzzaman A.S., Winnicki M., Lanfranchi P., Wolk R., Kara T., Accurso V., Somers V.K. (2002). Elevated C-Reactive Protein in Patients With Obstructive Sleep Apnea. Circulation.

[43] Testelmans D., Tamisier R., Barone-Rochette G., Baguet J.-P., Roux-Lombard P., Pépin J.-L., Lévy P. (2013). Profile of circulating cytokines: Impact of OSA, obesity and acute cardiovascular events. Cytokine.

[44] Pasceri V., Willerson J.T., Yeh E.T.H. (2000). Direct Proinflammatory Effect of C-Reactive Protein on Human Endothelial Cells. Circulation.

[45] Kokturk O., Ciftci T.U., Mollarecep E., Ciftci B. (2005). Elevated C-Reactive Protein Levels and Increased Cardiovascular Risk in Patients With Obstructive Sleep Apnea Syndrome. Int. Heart J..

[46] Lui M.M.S., Lam J.C.M., Mak H.K.F., Xu A., Ooi C., Lam D.C.L., Mak J.C.W., Khong P.L., Ip M.S.M. (2009). C-reactive protein is associated with Obstructive sleep apnea independent of visceral obesity. Chest.

[47] Akashiba T., Akahoshi T., Kawahara S., Majima T., Horie T. (2005). Effects of Long-term Nasal Continuous Positive Airway Pressure on C-reactive Protein in Patients with Obstructive Sleep Apnea Syndrome. Intern. Med..

[48] Imani M.M., Sadeghi M., Khazaie H., Emami M., Bahmani D.S., Brand S. (2020). Evaluation of Serum and Plasma Interleukin-6 Levels in Obstructive Sleep Apnea Syndrome: A Meta-Analysis and Meta-Regression. Front. Immunol..

[49] Lee L.-A., Chen N.-H., Huang C.-G., Lin S.-W., Fang T., Li H.-Y. (2010). Patients with severe obstructive sleep apnea syndrome and elevated high-sensitivity C-reactive protein need priority treatment. Otolaryngol. Neck Surg..

[50] Lee L.A., Huang C.G., Chen N.H., Wang C.L., Fang T.J., Li H.Y. (2011). Severity of Obstruc-tive Sleep Apnea Syndrome and High-Sensitivity C-Reactive Protein Reduced After Relocation Pharyngoplasty. Otolaryngol. Head Neck Surg..

[51] Garcia V.P., Rocha H.N.M., Sales A.R.K., Rocha N.G., da Nóbrega A.C.L. (2016). Diferenças na proteína c reativa ultrassensível associado ao gênero em indivíduos com fatores de risco da síndrome metabólica. Arq. Bras. Cardiol..

[52] Jung J.H., Park J.W., Kim D.H., Kim S.T. (2019). The Effects of Obstructive Sleep Apnea on Risk factors for Cardiovascular diseases. Ear Nose Throat J..

[53] Guilleminault C., Kirisoglu C., Ohayon M.M. (2004). C-Reactive Protein and Sleep-Disordered Breathing. Sleep.

[54] Bouloukaki I., Mermigkis C., Tzanakis N., Kallergis E., Moniaki V., Mauroudi E., Schiza S.E. (2017). Evaluation of Inflammatory Markers in a Large Sample of Obstructive Sleep Apnea Patients without Comorbidities. Mediat. Inflamm..

[55] Minoguchi K., Yokoe T., Tazaki T., Minoguchi H., Tanaka A., Oda N., Okada S., Ohta S., Naito H., Adachi M. (2005). Increased Carotid Intima-Media Thickness and Serum Inflammatory Markers in Obstructive Sleep Apnea. Am. J. Respir. Crit. Care Med..

[56] Greenberg H., Ye X., Wilson D., Htoo A.K., Hendersen T., Liu S.F. (2006). Chronic intermittent hypoxia activates nuclear factor-κB in cardiovascular tissues in vivo. Biochem. Biophys. Res. Commun..

[57] Kheirandish-Gozal L., Gozal D. (2019). Obstructive Sleep Apnea and Inflammation: Proof of Concept Based on Two Illustrative Cytokines. Int. J. Mol. Sci..

[58] Kapur V.K., Auckley D.H., Chowdhuri S., Kuhlmann D.C., Mehra R., Ramar K. (2017). Clinical practice guideline OSA American Academy. J. Clin. Sleep Med..

